# Physical activity and teacher burnout: parallel and serial mediation by mindfulness and spiritual wellbeing

**DOI:** 10.3389/fpsyg.2026.1788468

**Published:** 2026-04-08

**Authors:** Jia-Wei Yu, Xiang-Hui Liu, Xin Yi, Hou-An Zhao

**Affiliations:** 1 Department of Physical Education, Dankook University, Yongin-si, Republic of Korea; 2 Department of Exercise and Medical Science, Dankook University, Chungcheongnam-do, Republic of Korea; 3 Department of Pain, The First Affiliated Hospital, Hengyang Medical School, University of South China, Hengyang, China; 4 Department of Physical Education and Health, Changji University, Changji, China

**Keywords:** mindfulness, physical activity, serial mediation, spiritual wellbeing, teacher burnout

## Abstract

**Objective:**

Based on the job demands–resources model and conservation of resources theory, this study examines the association between physical activity and teacher burnout among primary and secondary school physical education teachers, focusing on the mediating roles of mindfulness and spiritual wellbeing.

**Methods:**

A cross-sectional design was employed to conduct a questionnaire survey among 313 primary and secondary school physical education teachers. Physical activity, mindfulness, spiritual wellbeing, and teacher burnout were assessed using validated scales. Hierarchical multiple regression and bootstrap-based serial mediation analysis (PROCESS Model 6) were employed to test the hypothesized serial mediation model.

**Results:**

The results showed that physical activity was associated with teacher burnout through three indirect pathways: (1) the mediating role of mindfulness (Effect = −0.046); (2) the mediating role of spiritual wellbeing (Effect = −0.038); and (3) the sequential mediating role of mindfulness and spiritual wellbeing (Effect = −0.039). The total indirect effect was −0.123.

**Discussion:**

The findings suggest that higher physical activity is associated with lower teacher burnout through both independent and sequential associations involving mindfulness and spiritual wellbeing. These findings should be interpreted cautiously, as the cross-sectional design does not permit causal inference. Nevertheless, the results highlight a potentially meaningful psychological resource pattern that may inform future intervention and longitudinal research.

## 1 Introduction

Commonly identified as a high-stress occupational group, teachers are subject to the prolonged demands of pedagogical instruction and classroom administration and exhibit distinct characteristics of emotional labor (Guo et al., 2022; Tsubono and Ogawa, 2022). To maintain emotional expressions consistent with their professional roles, teachers must continuously regulate their emotions, which steadily depletes their psychological resources (Isenbarger and Zembylas, 2006; Mérida-López et al., 2017). Existing studies have further identified administrative burdens, teaching pressures, and role conflicts as critical stressors that significantly increase educators' psychological vulnerability (Aloe et al., 2014; Brady and Wilson, 2021; Jomuad et al., 2021). Moreover, prolonged exposure to chronic high-demand environments without adequate recovery increases susceptibility to burnout, which is primarily characterized by emotional exhaustion, depersonalization, and reduced personal accomplishment (Martinsone et al., 2024; Maslach and Jackson, 1981). Teacher burnout extends beyond purely affective exhaustion, often entailing systemic damage manifested through somatic dysregulation, chronic physical fatigue, and impaired cognitive-psychological functioning (Gebel et al., 2022; Sánchez-Narváez et al., 2023). Therefore, identifying and strengthening psychological resources that exert a buffering effect in high-stress environments has emerged as a crucial research trajectory for understanding and intervening in teacher burnout.

Physical activity can simultaneously address physiological regulation, physical functioning, and psychological states, and regular physical activity may mitigate burnout-related allostatic loads by modulating cortisol secretion, autonomic nervous system balance, and systemic inflammatory response levels (Mincarone et al., 2024). Physiologically, physical activity facilitates physical conditioning and fatigue reduction while optimizing sleep architecture, effectively buffering the physical strain stemming from high job demands (Alnawwar et al., 2023). Psychologically, participation in physical activity is frequently accompanied by more positive affective experiences, heightened self-efficacy, and enhanced self-regulatory capacity, all of which are critical psychological correlates fundamentally linked to the core manifestations of burnout (White et al., 2024). Furthermore, Malagodi et al. (2024) validated the immediate facilitative effects of physical activity on emotional states, demonstrating that various types of moderate-intensity physical activity can enhance transient affective responses. Nevertheless, the psychological pathways linking physical activity to teacher burnout remain underexplored, and few studies have concurrently scrutinized the interplay between multiple mediators and their potential serial mediation effects.

To clarify the association between physical activity and teacher burnout through resource-related pathways and psychological mediators, the Job Demands–Resources (JD-R) model and the Conservation of Resources (COR) theory provide essential theoretical frameworks (Demerouti et al., 2001; Hobfoll, 1989). The JD-R model posits that high job demands lead to resource depletion and a health-impairment process, whereas the acquisition and accumulation of resources can buffer stress and reduce the risk of burnout (Bakker and Demerouti, 2007). COR theory further emphasizes that individuals can acquire new resources through resource investment, thereby enhancing their resilience to stress during the resource gain process (Hobfoll, 1989, 2001, 2002). In this study, physical activity may serve as a form of resource investment that is associated with teacher burnout through resource-based pathways, partly by supporting the generation and recovery of personal resources (Wang et al., 2025; Xu et al., 2025). To address this, it is useful to examine the potential psychological pathways linking physical activity and teacher burnout from a psychological perspective by incorporating mindfulness and spiritual wellbeing into the model as two key categories of psychological resources (Bickerton et al., 2015; Grover et al., 2017).

Mindfulness emphasizes present-moment awareness, attentional regulation, and orientation to experience, enabling individuals to maintain clearer self-awareness and more effective emotion regulation within high-stress environments (Bishop et al., 2004; Bunjak et al., 2022). Existing studies indicate that physical activity may provide critical support for the attentional and emotion regulation processes associated with mindfulness, thereby supporting stress regulation and present-moment awareness (Sars, 2022). Furthermore, regular physical activity is significantly correlated with higher levels of mindfulness (Ye et al., 2022; Zhao et al., 2024). Cross-occupational studies and teacher-specific studies reveal a robust inverse relationship between mindfulness and burnout (Green and Kinchen, 2021; Paudel et al., 2025). Empirical research further suggests that the higher the level of teacher mindfulness, the weaker the association between job demands and burnout (Guidetti et al., 2019; He et al., 2024). This indicates that mindfulness may serve as a protective buffer by enhancing the awareness and regulation of stress cues, which is in turn associated with lower burnout risk. Based on these premises, this study examines mindfulness as a critical psychological mediator in the association between physical activity and teacher burnout.

Spiritual wellbeing refers to an individual's subjective understanding and experience of meaning and purpose in life, as well as the resulting states of inner harmony and holistic subjective wellbeing (Ellison, 1983). As research suggests, regular physical activity provides consistency and concrete objectives for daily life, which can make it easier for individuals to cultivate a sense of purpose and direction and strengthen their experience of meaning (Yemiscigil and Vlaev, 2021). Furthermore, Luo et al. (2025) found that individuals with higher levels of physical activity tend to report greater spiritual wellbeing, providing empirical support for a positive association between physical activity and spiritual wellbeing. A study of secondary school teachers in Hong Kong found that all dimensions of spiritual wellbeing were negatively correlated with emotional exhaustion and depersonalization, and positively correlated with personal accomplishment (Pong, 2022). This suggests that spiritual wellbeing may serve as a critical psychological resource linked to teacher burnout. Based on this inference, spiritual wellbeing may function as a mediator in the association between physical activity and teacher burnout by helping individuals maintain psychological energy and sustained engagement and may therefore be associated with lower burnout.

In addition, research indicates that higher levels of mindfulness are typically associated with superior emotional regulation, internal awareness, and self-reflection capabilities (Palmer et al., 2023). This stable present-moment awareness helps individuals more clearly perceive their value orientations and life goals (Crego et al., 2021), which ultimately bolsters their sense of existential meaning (Hanner, 2024). These psychological transformations may provide one possible foundation for the development of spiritual wellbeing (Berna et al., 2024), offering preliminary support for a potential association between mindfulness and spiritual wellbeing within the broader relationship between physical activity and teacher burnout.

Drawing upon the aforementioned research, this study further examines the association between physical activity and teacher burnout while investigating the mediating roles of mindfulness and spiritual wellbeing as psychological resources. The proposed ordering between mindfulness and spiritual wellbeing should be understood as a theoretically informed sequence rather than a confirmed temporal process. Accordingly, the following hypotheses are proposed:

H1: Mindfulness mediates the association between physical activity and teacher burnout.

H2: Spiritual wellbeing mediates the association between physical activity and teacher burnout.

H3: Mindfulness and spiritual wellbeing sequentially mediate the association between physical activity and teacher burnout.

## 2 Materials and methods

### 2.1 Participants

In this cross-sectional study, primary and secondary school physical education teachers were randomly selected from schools in Xinjiang Province of China. All participants who received the link to the online questionnaire were informed about the study's purpose, content, and response procedures, and provided consent to participate. Participants were also explicitly encouraged to respond honestly. Out of 361 responses, 48 were excluded from the analysis due to missing answers or patterns indicating insincere responses, such as selecting the same option repeatedly. Therefore, the final analysis was conducted using 313 valid responses, yielding an effective response rate of 86.65%. In terms of gender, 83 participants were male (26.5%) and 230 were female (73.5%). Regarding age, 199 participants were 29 years or younger (63.6%), 74 were aged 30–39 years (23.6%), 32 were aged 40–49 years (10.2%), 5 were aged 50–59 years (1.6%), and 3 were aged 60 years or older (1.0%). For teaching level, 83 taught at the primary school level (26.5%), 84 at the middle school level (26.8%), and 146 at the high school level (46.6%). With respect to educational attainment, 21 participants had an associate degree or below (6.7%), 207 held a bachelor's degree (66.1%), and 85 held a postgraduate degree (27.2%). Regarding teaching experience, 184 participants reported 0–5 years (58.8%), 64 reported 6–10 years (20.4%), 39 reported 11–20 years (12.5%), 14 reported 21–30 years (4.5%), and 12 reported 31 years or more (3.8%).

## 3 Measures

### 3.1 Physical activity

Physical activity volume was assessed using the International Physical Activity Questionnaire–Short Form (IPAQ-SF). The IPAQ-SF asks respondents to recall their participation in physical activities of different intensities over the previous seven days, including vigorous activity, moderate activity, walking, and sitting time (Craig et al., 2003). Following the IPAQ-SF, participants reported the frequency (days/week) and duration (minutes/day) of each activity intensity. These values were weighted by the corresponding metabolic equivalent task (MET) values: walking (3.3 METs), moderate activity (4.0 METs), and vigorous activity (8.0 METs) to calculate physical activity volume. Physical activity volume for each intensity category was expressed as MET-min/week and calculated as MET value × frequency (days/week) × duration (minutes/day). Total physical activity volume was obtained by summing the MET-min/week values for walking, moderate activity, and vigorous activity. Sitting time was recorded as a separate indicator and was not included in the total physical activity volume score (IPAQ Research Committee, 2005).

### 3.2 Mindfulness

Mindfulness was assessed using the Toronto Mindfulness Scale (TMS) developed by Lau et al. (2006), with the validated Chinese version revised by Yu et al. (2021), to measure participants' mindfulness. TMS consists of 13 items and includes two subscales: curiosity and decentering, which assess individuals' modes of attending to present moment experience and the psychological distance they maintain from their internal experiences. All items are rated on a 5-point Likert scale, with higher scores indicating higher levels of mindfulness. In this study, this scale demonstrated excellent internal consistency (Cronbach's α = 0.944). Confirmatory factor analysis indicated an adequate model fit (RMSEA = 0.077, CFI = 0.983, SRMR = 0.022).

### 3.3 Spiritual wellbeing

Spiritual wellbeing was assessed using the Spirituality Scale revised by Ren et al. (2021) to measure participants' spiritual wellbeing. This scale consists of 12 items and includes two subscales: self-efficacy and meaning in life. It assesses individuals' functional self-efficacy in everyday life and their subjective perceptions of meaning in life. All items are rated on a 5-point Likert scale, with higher scores indicating higher levels of spiritual wellbeing. In this study, this scale demonstrated excellent internal consistency (Cronbach's α = 0.938). Confirmatory factor analysis indicated an adequate model fit (RMSEA = 0.074, CFI = 0.985, SRMR = 0.023).

### 3.4 Teacher burnout

Teacher burnout was assessed using the 10-item short form of the Maslach Burn-out Inventory (MBI) adopted by Liu et al. (2021), which was developed based on the original MBI (Maslach and Jackson, 1981). This scale comprises three subscales: emotional exhaustion, depersonalization, and reduced personal accomplishment. All items are rated on a 5-point Likert scale, with higher scores indicating higher levels of burnout. In this study, this scale demonstrated excellent internal consistency (Cronbach's α = 0.888). Confirmatory factor analysis indicated an adequate model fit (RMSEA = 0.059, CFI = 0.991, SRMR = 0.022).

## 4 Data processing

Data processing and statistical analyses were conducted using SPSS 26.0 (IBM Corp, Armonk, NY, USA). Harman's single-factor test was performed to provide an initial assessment of potential common method bias (CMB). Pearson correlation analyses were then conducted to examine bivariate associations among the study variables. Hierarchical multiple regression analysis and the PROCESS macro for SPSS (Hayes, 2013) were used to test the mediation effects, with Model 6 specified to examine the serial mediation pathways. Bias-corrected nonparametric percentile bootstrapping was used to generate confidence intervals for the mediation effects. The normality of the study variables was evaluated using the Kolmogorov–Smirnov test and the skewness and kurtosis statistics. Although the Kolmogorov–Smirnov test was significant (*p* < 0.05), the skewness (0.047 to 0.243) and kurtosis (−1.201 to −0.311) values suggested approximate normality. Because physical activity was measured in MET units, the physical activity variable was standardized prior to the mediation analyses to improve the comparability of coefficients.

## 5 Results

### 5.1 Common method bias

The first factor accounted for 35.80% of the total variance, which was below the conventional 40% threshold. This result provides only preliminary evidence that common method bias may not be severe; however, such bias cannot be fully ruled out in a cross-sectional self-report study.

### 5.2 Correlation analysis

Table 1 indicates the Pearson correlation coefficients among physical activity, mindfulness, spiritual wellbeing, and burnout. Physical activity was positively correlated with mindfulness (*R* = 0.401, *p* < 0.01) and spiritual wellbeing (*R* = 0.340, *p* < 0.01), and negatively correlated with burnout (*R* = −0.197, *p* < 0.01). Mindfulness was positively associated with spiritual wellbeing (*R* = 0.504, *p* < 0.01). In addition, both mindfulness (*R* = −0.319, *p* < 0.01) and spiritual wellbeing (*R* = −0.392, *p* < 0.01) were negatively correlated with burnout, which qualified for the mediation effects test.

**Table 1:** Correlation analysis of physical activity, mindfulness, spiritual wellbeing, and teacher burnout.

Variables	Mean	Standard deviation	1	2	3	4
Physical activity	2871.237	1590.306	1			
Mindfulness	3.017	0.854	0.401^**^	1		
Spiritual wellbeing	2.992	0.850	0.340^**^	0.504^**^	1	
Teacher burnout	2.987	0.770	−0.197^**^	−0.319^**^	−0.392^**^	1

^**^p < 0.01.

### 5.3 Serial mediation effect test

**Figure 1:**
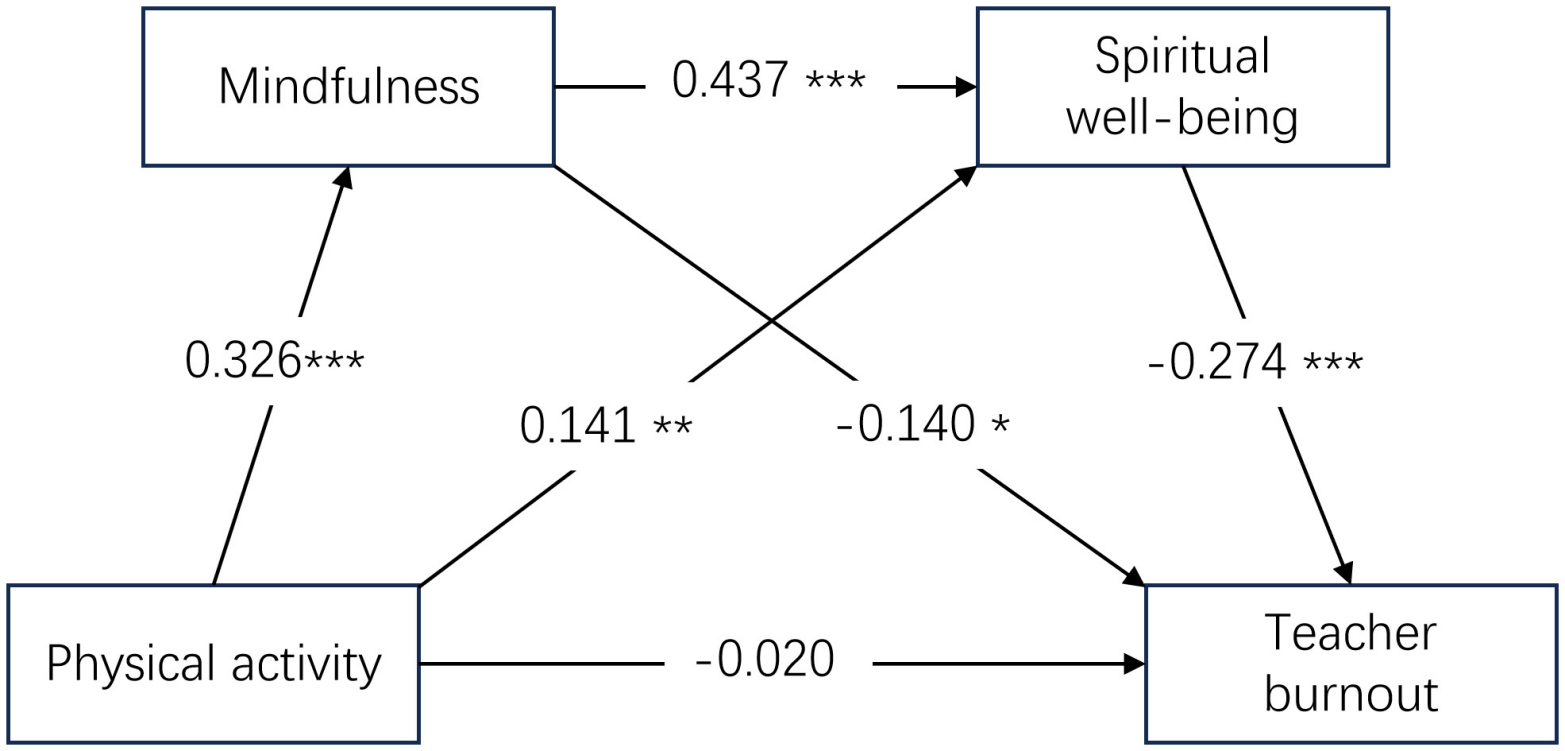
The serial mediating model of mindfulness and spiritual wellbeing. *p < 0.05, **p < 0.01, ***p < 0.001.

As shown in Figure 1, we conducted hierarchical multiple regression analyses while controlling for gender, age, teaching level, educational attainment, and teaching experience. Using physical activity as the independent variable and mindfulness as the dependent variable, the regression model was significant (*R*^2^ = 0.173, *F* = 10.649, *p* < 0.001). Physical activity had a significant positive effect on mindfulness (β = 0.326, *SE* = 0.046). When mindfulness was included in the regression equation predicting spiritual wellbeing (*R*^2^ = 0.287, *F* = 17.512, *p* < 0.001), physical activity (β = 0.141, *SE* = 0.046) and mindfulness (β = 0.437, *SE* = 0.053) positively predicted spiritual wellbeing. In addition, when burnout was entered as the dependent variable (*R*^2^ = 0.192, *F* = 9.034, *p* < 0.001), mindfulness (β = −0.140, *SE* = 0.057) and spiritual wellbeing (β = −0.273, *SE* = 0.055) negatively predicted teacher burnout, whereas the direct effect of physical activity on teacher burnout was not significant (β = −0.020, *SE* = 0.045).

We tested the significance of the mediating effect using the bootstrap method. As shown in Table 2, mindfulness (β = −0.046, 95% CI [−0.090, −0.008]) and spiritual wellbeing (β = −0.038, 95% CI [−0.073, −0.011]) not only played significant and independent mediating roles in the relationship between physical activity and teacher burnout, but also formed a significant serial mediating pathway (β = −0.039, 95% CI [−0.063, −0.021]).

**Table 2:** The serial mediation effect analysis.

Effect	β	SE	95% CI	Proportion
Total indirect effect	−0.123	0.026	[−0.177, −0.074]	100
Indirect effect 1	−0.046	0.021	[−0.090, −0.008]	37.40
Indirect effect 2	−0.038	0.016	[−0.073, −0.011]	30.90
Indirect effect 3	−0.039	0.011	[−0.063, −0.021]	31.70

Indirect effect 1 represents the indirect path: “physical activity-mindfulness-teacher burnout.” indirect effect 2 represents the indirect path: “physical activity-spiritual wellbeing-teacher burnout.” indirect effect 3 represents the indirect path: “physical activity–mindfulness–spiritual wellbeing–teacher burnout.”

## 6 Discussion

### 6.1 The mediating role of mindfulness

In this study, mindfulness mediated the association between physical activity and teacher burnout, which is consistent with findings from prior studies (Hidajat et al., 2023). Mindfulness, characterized by present-moment awareness, attentional regulation, and experiential orientation, may represent an important psychological pathway linking physical activity and teacher burnout (Bishop et al., 2004). Regular physical activity involves ongoing monitoring of physical states, sustained attentional focus, and adaptive responses to fatigue and negative emotions, which aligns with the core tenets of attention regulation and experiential orientation emphasized in mindfulness (Bazzano et al., 2018; Shahbaz and Sajjad, 2020). Furthermore, sustained aerobic exercise has been shown to significantly enhance mindfulness levels, providing direct empirical evidence for the role of physical activity in promoting mindfulness (Mothes et al., 2014). Additionally, mindfulness may help buffer the health-impairment process associated with burnout by altering individuals' appraisal of stress cues and their coping mechanisms (Keng et al., 2011). Specifically, mindfulness enables individuals to identify stress signals earlier and regulate them promptly, reducing rumination and persistent hyperarousal, thereby buffering emotional exhaustion (Gu et al., 2015). Meanwhile, non-judgmental acceptance reduces negative attributions and self-criticism, lowers defensive detachment, and thus curbs depersonalization, while sustaining feelings of competence and engagement that enhance personal accomplishment (Flook et al., 2013).

### 6.2 The mediating role of spiritual wellbeing

Spiritual wellbeing mediated the relationship between physical activity and teacher burnout in this study, consistent with the perspectives in spiritual psychology that regard spiritual wellbeing as a vital resource for stress adaptation (Chirico et al., 2020). The association between physical activity and spiritual wellbeing primarily arises from the cumulative sense of competence and meaningful experiences that are continuously built through physical activity (Aydin et al., 2020). Regular physical activities strengthen the sense of competence through goal setting, persistent investment, and feedback achievement, thereby reinforcing individuals' self-efficacy (Konopack and McAuley, 2012). Meanwhile, physical activities with contemplative attributes or nature connectedness are more likely to trigger reflection and meaning-making, supporting the sustained cultivation of a sense of purpose (Güllü, 2021). Furthermore, group-based physical activities provide social support through a sense of belonging and interpersonal connection, establishing a relational foundation for meaning-making (Austin, 2010). Spiritual wellbeing may buffer the health-impairment process associated with burnout by helping maintain an individual's meaning framework and self-efficacy (Oglesby et al., 2021). By fostering value congruence and vocational calling alongside self-efficacy, teachers can prevent job stressors from being perceived as chronic resource depletion, which in turn mitigates emotional exhaustion and depersonalization and sustains a sense of personal accomplishment (Whitehead et al., 2023). Studies on the teaching profession have also shown that spiritual-related dimensions are systematically associated with core aspects of burnout, providing empirical evidence for the protective role of spiritual wellbeing (Vem et al., 2023). Consequently, the mediation of spiritual wellbeing highlights that the preventive function of physical activity is reflected not only in the mitigation of stress responses but also in the support and consolidation of meaning and efficacy resources.

### 6.3 The serial mediating role of mindfulness and spiritual wellbeing

This study identified a theoretically informed serial mediation pathway in which physical activity was associated with teacher burnout through mindfulness and spiritual wellbeing in sequence. Regular physical activity may enhance interoceptive awareness and attentional stability, making it easier for individuals to enter a state of mindfulness (Tihanyi et al., 2016). In contexts of high job demands, teachers may be more likely to process work experiences in an automatic and emotionally involved manner, which constricts the space for awareness and regulation and may contribute to exhaustion and a diminished sense of meaning (Hakanen et al., 2006). Mindfulness may provide an alternative way of processing stress, enabling individuals to perceive stress cues in a more decentered manner and thereby creating greater space for reflection and regulation (Shapiro et al., 2006). Such shifts in how individuals perceive their experiences may support deeper meaning-making and value integration, which may in turn contribute to the development of spiritual wellbeing (Garland et al., 2015; Gecht et al., 2014). When spiritual wellbeing is conceptualized as an integrative resource encompassing life meaning and self-efficacy, it may function as a psychological buffer against the health-impairment process by helping sustain engagement through the preservation of a meaning framework and self-efficacy, and is thus associated with lower burnout (Chirico et al., 2023). Importantly, because the present data are cross-sectional, this sequence should be interpreted as a theoretically informed ordering rather than a confirmed temporal chain. Alternative pathways, including reversed or reciprocal relations between mindfulness and spiritual wellbeing, remain plausible and should be examined in future longitudinal or experimental research.

## 7 Limitations and implications

The serial mediation results of this study provide a more process-oriented perspective on the association between physical activity and teacher burnout, suggesting that this relationship may be reflected less in a direct association and more in linked psychological resources. Physical activity may be associated with greater mindfulness, which may help teachers process stress experiences in a less automatic and more decentered manner, thereby supporting stress regulation (Isoard-Gautheur et al., 2019). Higher mindfulness may in turn be associated with greater spiritual wellbeing, helping teachers integrate stress experiences into a broader framework of meaning and self-efficacy, which may support sustained engagement (Petchsawang and McLean, 2017). These findings offer a more refined account of how psychological resources may be linked and coordinated within the JD-R framework, contributing to a more process-oriented understanding of resource generation, transformation, and integration (Bakker et al., 2023). Accordingly, teacher health promotion may benefit from a combined strategy that uses sustainable physical activity as a practical entry point while integrating mindfulness-based support and meaning-oriented resources to strengthen teachers' internal resource framework; although the indirect effects observed in this study were modest in magnitude, such effects should not be interpreted as trivial, because teacher burnout is a complex outcome influenced by multiple personal and contextual determinants, and even relatively small effects may accumulate into meaningful practical benefits when the target behavior is feasible, low-cost, and scalable across school settings.

This cross-sectional study has several limitations. First, because the study relied on cross-sectional self-report data, the serial mediation model should be interpreted as a theoretically informed pattern of associations rather than a definitive causal chain. Future research could strengthen causal inference through longitudinal tracking, diary studies, or randomized controlled/quasi-experimental designs. Second, physical activity was estimated as MET values via retrospective self-report questionnaires, which may be subject to recall bias and social desirability bias and have limited capacity to distinguish between different activity contexts. Future studies could integrate wearable devices and ecological momentary assessment (EMA) to enhance measurement precision and examine the differential effects of various physical activity contexts. In addition, because all focal variables were collected from the same source at a single time point, common method variance remains a concern. Although Harman's single-factor test was used as a preliminary diagnostic procedure, this approach cannot fully exclude common method bias. Third, the current model did not directly incorporate school-level job demands and organizational resources, nor did it explicitly account for the hierarchical structure of teachers nested within schools. Future studies could utilize multilevel modeling (MLM) to identify the boundary conditions of the serial mediation pathway.

## 8 Conclusions

This study provides a more nuanced understanding of the association between physical activity and teacher burnout through the interplay of psychological resources. The findings support both the parallel and serial mediating roles of mindfulness and spiritual wellbeing, suggesting that these constructs may represent linked psychological resources within the relationship between physical activity and teacher burnout. Given the cross-sectional design, these findings should be interpreted as theoretically informed associations rather than confirmed causal pathways. The findings suggest that programs aimed at promoting teacher wellbeing may benefit from using regular physical activity as a practical entry point, complemented by mindfulness-based support and meaning-oriented resources.

## Data Availability

The raw data supporting the conclusions of this article will be made available by the authors, without undue reservation.
